# Regional homogeneity changes between heroin relapse and non-relapse patients under methadone maintenance treatment: a resting-state fMRI study

**DOI:** 10.1186/s12883-016-0659-3

**Published:** 2016-08-18

**Authors:** Haifeng Chang, Wei Li, Qiang Li, Jiajie Chen, Jia Zhu, Jianjun Ye, Jierong Liu, Zhe Li, Yongbin Li, Ming Shi, Yarong Wang, Wei Wang

**Affiliations:** 1 Department of Radiology, Tangdu Hospital, the Fourth Military Medical University, Xi’an, Shaanxi 710038 China; 2 Department of Neurology, Xijing Hospital, the Fourth Military Medical University, Xi’an, Shaanxi 710038 China

**Keywords:** Methadone maintenance treatment, Heroin relapse, Craving, Regional homogeneity, fMRI

## Abstract

**Background:**

Methadone maintenance treatment (MMT) is recognized as one of the most effective treatments for heroin addiction but its effect is dimmed by the high incidence of heroin relapse. However, underlying neurobiology mechanism of heroin relapse under MMT is still largely unknown. Here, we took advantage of a resting-state fMRI technique by analysis of regional homogeneity (ReHo), and tried to explore the difference of brain function between heroin relapsers and non-relapsers in MMT.

**Methods:**

Forty MMT patients were included and received a 12-month follow-up. All patients were given baseline resting-state fMRI scans by using a 3.0 T GE Signa Excite HD whole-body MRI system. Monthly self-report and urine test were used to assess heroin relapse or non-relapse. Subjective craving was measured with visual analog scale. The correlation between ReHo and the degree of heroin relapse was analyzed.

**Results:**

Compared with the non-relapsers, ReHo values were increased in the bilateral medial orbitofrontal cortex, right caudate, and right cerebellum of the heroin relapsers while those in the left parahippocampal gyrus, left middle temporal gyrus, right lingual gyrus, and precuneus were decreased in heroin relapsers. Importantly, altered ReHo in the right caudate were positively correlated with heroin relapse rates or subjective craving response.

**Conclusions:**

Using the resting-state fMRI technique by analysis of ReHo, we provided the first resting-state fMRI evidence that right caudate may serve as a potential biomarker for heroin relapse prediction and also as a promising target for reducing relapse risk.

## Background

Drug addiction is a kind of chronic cerebral dysfunction [1]. Unlike cocaine and cannabis popular in foreign countries, heroin is popular in China [2]. Compared with cocaine and cannabis, heroin causes more tolerance and addiction. More seriously, once addicted with heroin, it is hard to abstinence [3]. Currently, methadone maintenance treatment (MMT) is a main strategy for the treatment of heroin addiction [4]. However, unfortunately, MMT cannot completely solve the problem of heroin abuse due to the fact that the relapse rate still remained high under MMT [2]. Apart from psychological and social factors, many researchers believed that neurobiological factors were also involved in heroin addiction and relapse [5].

In recent years, a body of studies have been making the attempt on neural circuits and brain functions in heroin addiction by using resting-state fMRI method. By comparing heroin addicts with healthy controls, researchers revealed a heroin-related organization pattern. For instance, Ma et al. [6] observed the enhanced functional connectivity between the nucleus accumbens and the ventral cingulate cortex, the orbitofrontal cortex (OFC), or the amygdala whereas the decreased functional connectivity between the anterior cingulate and the prefrontal cortex or the OFC was evident in heroin addicts. With the graph theory analysis, Liu et al. [7] found dysfunctional connectivity in the anterior cingulate cortex, amygdala, insula, hippocampus, nucleus accumbens, prefrontal cortex, supplementary motor area, and ventral striatum in chronic heroin users. Therefore, resting-state fMRI method could not only reveal the abnormal baseline function in heroin users at the resting state, but also show the abnormal functional connectivity of two remote areas.

Recently, the regional homogeneity (ReHo) method [8] has been developed to analyse the similarities or coherence of intraregional spontaneous low-frequency (<0.08 Hz) blood oxygenation level-dependent signal fluctuations in voxel-wise analysis across the whole brain. Due to independence of the onset time of stimulus, this method is useful for resting-state fMRI data analysis to indicate the resting state in a given brain region. It has been used to investigate the functional modulations in the resting state in healthy aging subjects [9] and in patients with Alzheimer disease [10], Parkinson disease [11], schizophrenia [12], and neuromyelitis optica [13]. Importantly, using this method, Qiu et al. [14] revealed that ReHo values in the bilateral OFC and medial thalamic of heroin addicts were lower than that in healthy subjects. However, at present we still lacked the knowledge about the changes of ReHo between heroin relapse and non-relapse patients receiving MMT.

In the present study, we included forty heroin addicts, and by using the resting-state fMRI technique to investigate the difference of resting-state brain function between heroin relapser and non-relapser in MMT patients by analysis of ReHo changes, trying to provide a further insight into the neurobiological mechanism underlying heroin relapse in MMT patients.

## Methods

### Participants

Sixty male former heroin addicts were recruited from the outpatient of the Baqiao Methadone Maintenance Treatment Center in Xi’an, Shaanxi Province, China. Patients were eligible for enrollment if they (a) met the diagnostic criteria of DSM-IV (Diagnostic and Statistical Manual of Mental Disorders, Fourth Edition) for heroin addiction; (b) willingly received long-term and stable MMT; (c) received at least three-month MMT; (d) were right-handed as judged by the Edinburgh Handedness Inventory [15]. Patients were excluded if they (a) had a history of active neurological and psychiatric disorders besides heroin addiction, (b) had a history of head trauma, medical disorder requiring immediate treatment, and contraindications to MRI examination.

Additionally, co-morbidity of addiction and psychiatric disorders is very common in heroin addicts, and to avoid the potential influence of psychiatric symptoms, we excluded the patients who reported having a current or past psychiatric illness other than heroin addiction, as described in exclusion criteria. We also evaluated the baseline psychological problems which usually accompanied with heroin addiction, such as depression and anxiety. The Beck Depression Inventory II and Hamilton Anxiety Scale were used to evaluate the severity of depression and anxiety symptoms respectively. There were no significant differences in depression severity and anxiety level between groups (data not shown).

This study was approved by Institutional Board of the Fourth Military Medical University, Xi’an, China and conducted in accordance with Declaration of Helsinki. All participants were fully informed about the details of experiment and signed the written consents for their involvement and The proposed protocol conforms to the Good Clinical Practice and has been approved by the Ethics Committee of the Institute of Tangdu Hospital, Fourth Military Medical University, Shaanxi, China.

### Experiment design and procedure

There were 60 participants recruited in the study. According to the inclusion criteria exclusion criteria, 20 participants were excluded for having other active neurological and psychiatric disorders, head trauma, or contraindications to MRI examination, and 40 were included and then received a baseline resting-state fMRI scanning following with a succeeding 12-month clinical follow-up. During the follow-up, they were supervised under monthly structured interview and urine tests. The participants with once self-reported heroin use or/and positive urine tests were defined as heroin relapser, and those with negative results in tests were defined as non-relapser. Relapse rate of each heroin relapser was calculated as following: total numbers of positive results in tests during MMT/12. The subjective heroin craving was assessed by a 0–10 score visual analogue scale (VAS).

### Image acquisition

All MRI scans were conducted on a Signa EXCITEHD 3.0 T scanner (GE Healthcare, Milwaukee, USA) with an eight-channel head coil. A routine T_2_WI structural scan (parameters: TR = 5100 ms, TE = 117 ms, matrix = 416 × 416, FOV = 24 × 24 cm^2^, slice thickness = 5.5 mm, gap = 0.8 mm) was performed to exclude gross cerebral pathology and then a BOLD functional imaging data was acquired using T2*-weighted gradient-echo planar imaging pulse sequence (GE-EPI, 32 axial slices covering the whole brain, 150 volumes). The parameters were set as follows: TR = 2000 ms, TE = 30 ms, flip angle = 90°, matrix = 64 × 64, FOV = 256 × 256 mm^2^, slice thickness = 4 mm, gap = 0 mm; spatial resolution = 4 × 4 × 4 mm^3^. The corresponding high-resolution fast spoiled gradient-echo 3D T_1_-weighted images were also collected for anatomical overlays of the functional data and for spatial normalization of the datasets to a standard atlas and settings were as following: TR = 7.8 ms, TE = 3.0 ms, matrix = 256 × 256, FOV = 256 × 256 mm^2^, spatial resolution =1 × 1 × 1 mm^3^.

### Data processing and regional homogeneity calculation

Data preprocessing was performed with software (SPM12; http://www.fil.ion.ucl.ac.uk/spm) as described previously [14]. In brief, for each participant the first 10 time points were discarded to avoid transient signal changes before magnetization reached steady state. Translation and rotation were checked and subjects with head movements exceeding than 1 mm in any direction or head rotations greater than 1° were discarded. After the motion correction, spatial normalization was made to the Montreal Neurological Institute (MNI) template (resampling voxel size = 3 × 3 × 3 mm^3^). The T1-weighted high-resolution image volume was also spatially normalized to the MNI template. All images spatially normalized to MNI template were transformed to Talairach and Tournoux coordinates and then filtered by using a band-passed filter (0.01–0.08 Hz) to reduce the effect of low-frequency drifts and high-frequency noise. All the data was collected and analyzed by an author (Y.B.L., 5 years of experience in fMRI analysis) who was blinded to the present study.

For regional homogeneity (ReHo) analysis, the data were processed with a software (REST1.8; http://resting-fmri.sourceforge.net). The ReHo calculation procedure was performed as previously reported [8]. In brief, it was performed on a voxel-by-voxel basis by calculating the Kendall Coefficient of Concordance (KCC) of the time series of a given voxel and that of its nearest neighbors. To reduce the influence of individual variation in the KCC, ReHo maps were normalized to the KCC of each voxel by the averaged KCC of the whole brain. The resulting data were then smoothed spatially with a 6-mm full width at half maximum Gaussian kernel to reduce noise and residual differences in gyral anatomy.

### Statistical analysis

To analyse the differences of ReHo values between heroin relapse and non-relapse patients under MMT, a two-sample *t* test was performed on the individual normalized ReHo maps in a voxel-by-voxel manner. A probability of *p* < 0.05 (corrected by FDR) was considered statistically significant.

### Correlation analysis

To evaluate the association of altered ReHo in different brain regions with heroin relapse in these relapsers, we performed Spearman's correlation analysis between mean ReHo values and relapse rates (as defined above) or subjective heroin craving indicated by VAS scores of each heroin relapser. The correlation analysis was based on non-parametric Spearman correlations and included only the relapsers. Threshold levels of significance for correlation coefficients were adjusted for multiple comparisons by a Bonferroni’s correction (*p* value was set as 0.05, and the number of tests was 8).

## Results

Sixty male former heroin addicts were recruited in the present study. Due to head motion, abnormal brain structure and incomplete outcome data, forty were finally included according to the inclusion and exclusion criteria. After 12-month follow-up, 21 participants were classified as relapsers according to the defined criteria for relapse, and 19 as non-relapsers. There were no significant differences between heroin relapsers and non-relapsers in their age, education, number of cigarettes smoked per day, duration of cigarette smoking, duration of heroin abuse and daily/accumulated dosage of heroin/methadone (Table 1).

**Table 1:** Clinical characteristics of participants (mean ± S.D.)

Characteristics	Non-relapse (*N* =19)	Relapse (*N* = 21)	T-value	*p*-value
Age (years)	39.3 ± 7.1	35.6 ± 5.4	1.74	0.09
Education level (years)	9.1 ± 2.6	9.5 ± 2.2	−0.49	0.62
Cigarettes (per day)	21 ± 8.5	21.3 ± 10.1	−0.12	0.90
Duration of cigarette smoking (months)	275.7 ± 69.1	224 ± 64.5	2.29	0.30
Daily dosage of heroin abuse (g)	0.3 ± 0.2	0.3 ± 0.1	0.16	0.87
Accumulated dosage of heroin abuse (g)	1036.8 ± 1232.2	918.8 ± 1186.9	0.28	0.77
Duration of heroin abuse (months)	275.7 ± 69.1	224 ± 64.5	0.49	0.62
Accumulated dosage of methadone use (l)	41.6 ± 34.9	30.6 ± 17.4	1.16	0.25
Daily dosage of methadone use (ml)	44.1 ± 19.5	43.5 ± 11.8	0.11	0.91

*Note*: Unless otherwise indicated, data are means ± standard deviations (S.D.)

**Fig. 1:**
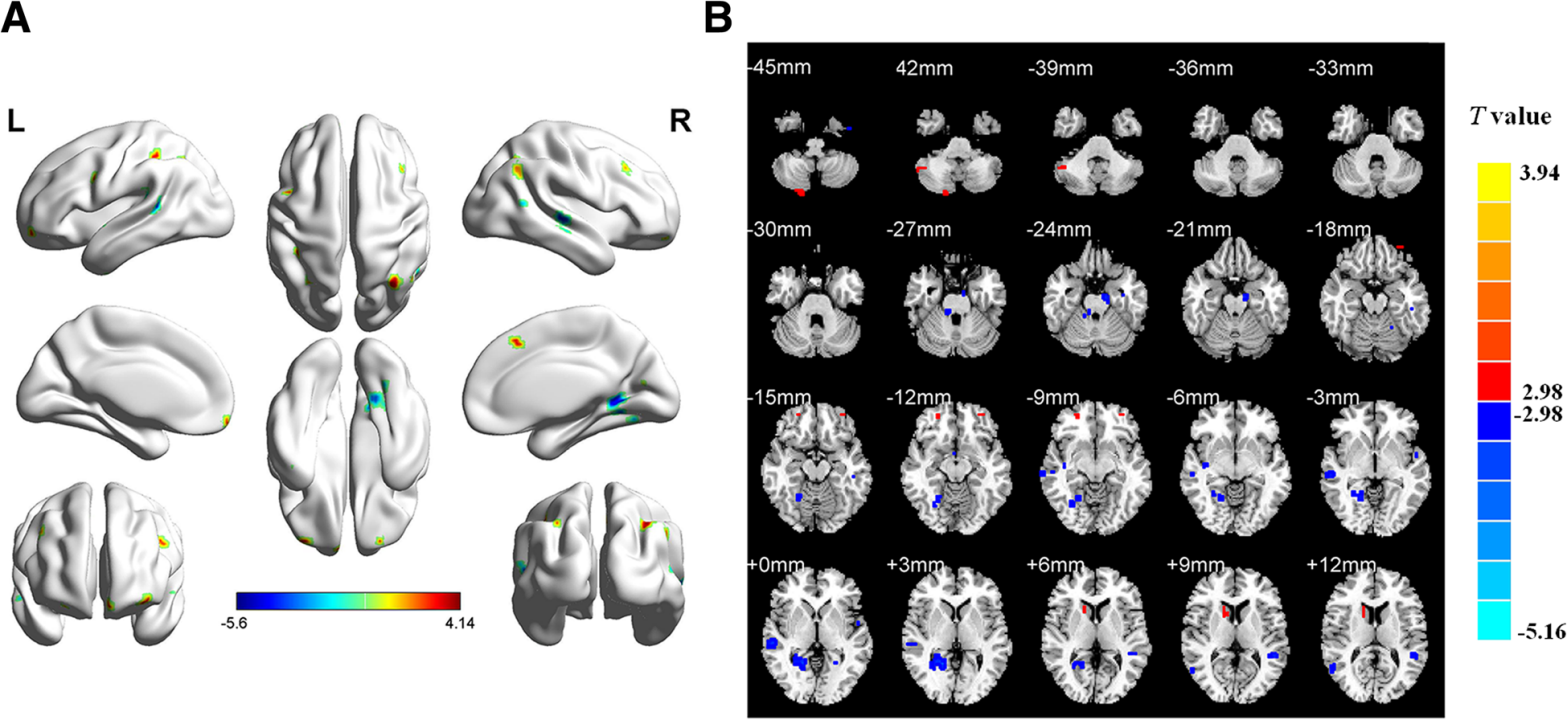
Data show brain areas with abnormal ReHo in heroin relapsers compared with non-relapsers under MMT. The differences are shown on whole-brain MR rendering (**a**) and MR axial view (every 3 mm, **b**). Relapsers displayed increased ReHo in the bilateral medial OFC, right caudate, and right cerebellum (*red*) but decreased ReHo in the left parahippocampal gyrus, left middle temporal gyrus, right lingual gyrus, and precuneus (*blue*). (*P* < 0.05, corrected)

Compared with heroin non-relapsers, the relapsers showed significant increases of ReHo in the bilateral medial OFC, right caudate nucleus, and right cerebellum bilateral, but significant decreases of ReHo in the left parahippocampal gyrus, right lingual gyrus, right precuneus, and left middle temporal gyrus (Fig. 1 and Table 2).

**Table 2:** Brain regions with abnormal ReHo in heroin relapse and non-relapse patients under MMT

Brain area	Side	Mean ReHo		BA	MNI coordinate (mm)			Voxels	T value (voxel-level)
		Non-relapser	Relapser		x	y	z		
Orbitofrontal cortex	R	0.32 ± 0.49	0.32 ± 0.76	11	20	54	12	18	3.28
Orbitofrontal cortex	L	−0.20 ± 0.17	−0.01 ± 0.20	11	−33	54	18	16	3.25
Caudate nucleus	R	0.60 ± 0.53	1.10 ± 0.31	40	15	12	9	32	3.68
Cerebellum	R	−0.97 ± 0.64	−0.27 ± 0.62	22	45	−51	−39	22	3.48
Parahippocampal gyrus	L	−0.83 ± 0.58	−1.57 ± 0.55	34	−12	−12	−24	7	−4.11
Lingual gyrus	R	0.60 ± 0.51	−0.20 ± 0.56	19	21	−51	3	51	−4.68
Precuneus	R	0.60 ± 0.51	−0.20 ± 0.56	18	21	−51	3	10	−4.68
Middle temporal gyrus	L	0.20 ± 0.40	−0.44 ± 0.61	13	−51	−42	9	26	−3.89

*Note*: *R* right, *L* left, *BA* Brodmann area, *MNI* Montreal Neurological Institute

We then analyzed the association of altered ReHo values in the identified regions (including bilateral medial OFC, right caudate, right cerebellum, left parahippocampal gyrus, left middle temporal gyrus, right lingual gyrus, and right precuneus) with relapse rates or with subjective heroin craving in relapse patients. Our results showed a positive correlation was observed between mean ReHo values in the right caudate and relapse rates or heroin craving whereas no correlation relationships were found between ReHo values and other brain regions (Tables 3 and 4). Additionally, we also made the correlation between the amount of motion in each subject and the ReHo values, and found a negative result (data not shown), indicating that head motion was not biasing the ReHo.

**Table 3:** Spearman correlation between mean ReHo of abnormal brain regions and relapse rate in relapse patients

Brain area	Side	MNI Coordinate (mm)			Correlation coefficient	Corrected *p* value
		x	y	z		
Orbitofrontal cortex	R	20	54	12	−0.169	0.084
Orbitofrontal cortex	L	−33	54	18	−0.176	0.056
Caudate nucleus	R	15	12	9	0.380	0.018*
Cerebellum	R	45	−51	−39	−0.040	0.108
Parahippocampus gyrus	L	−12	−12	−24	−0.099	0.083
Lingual gyrus	R	21	−51	3	0.012	0.120
Precuneus	R	21	−51	3	0.014	0.106
Middle temporal gyrus	L	−51	−42	9	−0.076	0.093

*Note*: *R* right, *L* left, *BA* Brodmann area, *MNI* Montreal Neurological Institute

The significance for correlation coefficients was corrected by Bonferroni’s test. *p* value was set as 0.05. *, *p* < 0.05

**Table 4:** Spearman correlation between mean ReHo of abnormal brain regions and subjective heroin craving in relapse patients

Brain area	Side	MNI coordinate (mm)			Correlation coefficient	Corrected *p* value
		x	y	z		
Orbitofrontal cortex	R	20	54	12	−0.038	0.109
Orbitofrontal cortex	L	−33	54	18	−0.101	0.066
Caudate nucleus	R	15	12	9	0.395	0.010*
Cerebellum	R	45	−51	−39	0.082	0.078
Parahippocampus gyrus	L	−12	−12	−24	0.056	0.101
Lingual gyrus	R	21	−51	3	−0.093	0.086
Precuneus	R	21	−51	3	−0.108	0.071
Middle temporal gyrus	L	−51	−42	9	0.020	0.116

*Note*: *R* right, *L* left, *BA* Brodmann area, *MNI* Montreal Neurological Institute

The significance for correlation coefficients was corrected by Bonferroni’s test. *p* value was set as 0.05. *, *p* < 0.05

## Discussion

In this study we carried out a 12-month follow-up study using resting-state fMRI by analysis of brain regions with abnormal ReHo in heroin relapsers under MMT, and found that compared with the non-relapsers, an increased ReHo values were evident in the bilateral medial OFC, right caudate nucleus, and right cerebellum of the heroin relapsers while a decreased ReHo in the left parahippocampal gyrus, left middle temporal gyrus, right lingual gyrus, and precuneus. Moreover, ReHo values in right caudate nucleus were correlated with relapse rates or subjective heroin-craving response.

The medial OFC is a part of the limbic system, and is a functionally heterogeneous region that involved in complex adaptive behaviors. It was reported that in active cocaine abusers, OFC was hypermetabolic in proportion to the intensity of the craving experienced by the subjects [16] but was hypoactive in drug-addicted subjects tested during withdrawal [17]. Increased OFC activation has also been associated with drug-related cues [18], and compulsive drug intake [19, 20]. Importantly, a preclinical study showed that damage to the OFC resulted in a behavioral compulsion to procure the reward even when it is no longer reinforced [21]. In the present study, we found increased ReHo in the bilateral medial OFC in the heroin relapsers under MMT, indicating an enhanced local synchronization of spontaneous low-frequency blood oxygenation level-dependent fluctuations in this region, and probably reflecting abnormalities of relapsers in the affective value of reinforcers, decision making and expectation.

Caudate nucleus (dorsal striatum) is a principal brain region of mesostriatum dopamine (DA) pathway and is recognized to contribute to drug addiction [22]. A group of study have demonstrated that in the caudate nucleus existed significant DA changes when addicts were exposed to drug-related cues, and the magnitude of these changes were correlated with self-reports of craving [5, 23–25]. For the drug-dependent patients, the enhanced activity in the caudate in response to drug-related cues indicated increased reward-based cognitive processes in the presence of the cues [26]. These studies disclosed that the caudate nucleus may be a crucial link between conditional and behavioral responses to procure drug and the DA activity responding to drug-related cues, which mediates the habitual nature of subjective experience of caving and compulsive drug-seeking behaviors in addicts [27, 28]. In this study, we found that heroin relapsers’ ReHo values in right caudate were higher than non-relapsers, suggesting that relapsers had an abnormal reward response relative to the non-relapser even in the resting-sate.

Another area of the brain with increased ReHo in the present study was the cerebellum, which is reportedly involved in addictive behavior. Previous PET and fMRI studies revealed that drug-conditioned cue could activate the cerebellum and increased its metabolism [29, 30]. When addicts performed reward expectation tasks, glucose metabolism in their cerebellum was greatly increased [31]. These results suggested the cerebellum was involved in drug-conditioned memories in addicts. Since cerebellum and caudate are the primary site of rewarding feeling, increased ReHo values in these areas in the relapsers under MMT probably indicated an abnormal enhancement of rewarding effect on heroin or heroin-related cues, leading to compulsive drug-seeking behaviors and ultimately increasing the possibility of heroin relapse. Additionally, cerebellum is also related to learning and memory, increased ReHo in cerebellum may result in abnormal enhancement of positive or negative emotions during drug addiction, which in this study, may induce the occurrence of heroin relapse behaviors.

In contrast, ReHo in several brain areas, especially in precuneus, were significantly decreased in heroin relapsers relative to non-relapsers. Apart from extension of the visuo-spatial processes subserved by the lateral parietal cortices, precuneus also plays an important role in a diverse array of highly integrated functions, e.g., involving in voluntary attention shifts between targets and playing a central role in the modulation of conscious process [32]. Precuneus could integrate information, which related to drug cues in the environment with the previously learned association involving those cues and relaying that information to the prefrontal cortex [33]. We still lacked the knowledge about the decreased ReHo in precuneus of MMT relapsers. This may be associated with the destruction or clinical deficits of precuneus-related visual spatial attention and consciousness in heroin relapsers.

Moreover, we observed a positive correlation between altered ReHo values in the right caudate and relapse rates or heroin craving. These findings support the idea that heroin relapse and craving were related to the caudate nucleus, which plays an important role in the habitual nature of subjective experience of caving and compulsive drug-seeking behaviors in drug addicts [27, 28]. Importantly, it also suggests that right caudate nucleus may not only serve as a reliable biomarker for predicting heroin relapse but also as a promising target for the treatment of MMT relapse.

Admittedly, some limitations in the present are worth noting. First, in this study, female subjects were not included. Thus, a possible gender difference in relapse susceptibility was unexplored. Second, the interval of monthly self-report and urine test to assess MMT relapse was relatively long, probably not reflecting the true relapse rates. Finally, it had certain limitations by using urine test to assess the severity of MMT relapse. In addition, in spite of the results obtained above, we still lacked the knowledge about which neural circuit played the crucial role in the relapse behavior in heroin addicts receiving MMT. Therefore, other analysis methods such as fractional amplitude of low frequency fluctuations, degree of centrality maps, and the other metrics provided by resting-state fMRI should be employed to analyze the relationship of resting functional characteristics and relapse need further exploration.

## Conclusion

In summary, compared with non-relapsers, heroin relapsers had an abnormal resting-state function. Specially, relapsers displayed increased ReHo in the bilateral medial OFC, right caudate, and right cerebellum but decreased ReHo in the left parahippocampal gyrus, left middle temporal gyrus, right lingual gyrus, and precuneus. The alterations of ReHo in these areas may involve abnormal motivation, reward, memory and visual spatial attention. Overall, our results may add to neurobiological factors associated with relapse to heroin use and also be conducive to developing a promising strategy for heroin addiction therapies.

## Abbreviations

MMT, methadone maintenance treatment; OFC, orbitofrontal cortex; ReHo, regional homogeneity; VAS, visual analogue scale
